# Unidentified *CYP2D6* genotype does not affect pharmacological treatment for patients with first episode psychosis

**DOI:** 10.1177/02698811241279022

**Published:** 2024-09-29

**Authors:** Emma Y De Brabander, Thérèse van Amelsvoort, Roos van Westrhenen, GROUP Investigators

**Affiliations:** 1 Department of Psychiatry and Neuropsychology, Mental Health and Neuroscience Research Institute, Maastricht University Medical Centre, The Netherlands; 2 Department of Psychiatry, Parnassia Groep BV, The Netherlands; 3 Institute of Psychiatry, Psychology and Neurosciences, King’s College London, London, UK; 4 St. John’s National Academy of Health Sciences, Bangalore, India

**Keywords:** Pharmacogenetics, *CYP2D6*, drugs for psychosis, psychosis

## Abstract

**Background:**

Research on the pharmacogenetic influence of hepatic *CYP450* enzyme 2D6 (*CYP2D6*) on metabolism of drugs for psychosis and associated outcome has been inconclusive. Some results suggest increased risk of adverse reactions in poor and intermediate metabolizers, while others find no relationship. However, retrospective designs may fail to account for the long-term pharmacological treatment of patients. Previous studies found that clinicians adapted risperidone dose successfully without knowledge of patient *CYP2D6* phenotype.

**Aim:**

Here, we aimed to replicate the results of those studies in a Dutch cohort of patients with psychosis (*N* = 418) on pharmacological treatment.

**Method:**

We compared chlorpromazine-equivalent dose between *CYP2D6* metabolizer phenotypes and investigated which factors were associated with dosage. This was repeated in two smaller subsets; patients prescribed pharmacogenetics-actionable drugs according to published guidelines, and risperidone-only as done previously.

**Results:**

We found no relationship between chlorpromazine-equivalent dose and phenotype in any sample (complete sample: *p* = 0.3, actionable-subset: *p* = 0.82, risperidone-only: *p* = 0.34). Only clozapine dose was weakly associated with *CYP2D6* phenotype (*p* = 0.03).

**Conclusion:**

Clinicians were thus not intuitively adapting dose to *CYP2D6* activity in this sample, nor was *CYP2D6* activity associated with prescribed dose. Although the previous studies could not be replicated, this study may provide support for existing and future pharmacogenetic research.

## Background

Failure to respond to pharmacological treatment remains a serious concern for psychosis and psychotic disorders including schizophrenia. Although drugs for psychosis are reported to be successful in reducing symptoms compared to placebo (Huhn et al., 2019), the process of finding medication with optimal desired results and minimal adverse reactions can be long and challenging. Even when symptom improvement is established, adverse reactions such as extrapyramidal symptoms, dry mouth, dizziness, or weight gain associated with drugs for psychosis may affect patient quality of life and discourage adherence to medication (Acosta et al., 2012; Ayre et al., 2023). Increasing numbers of publications suggest that there are ways to improve prescription of pharmacological treatment by patient-tailored prescription (Arranz and de Leon, 2007; Płaza et al., 2022; van Westrhenen et al., 2021; Zhang and Malhotra, 2011).

One area of personalized medicine is pharmacogenetics (PGx). PGx covers the field of studying genes associated with medication metabolism, most notably the hepatic cytochrome P450 (CYP) enzymes in this context, as well as non-pharmacokinetic genes involved in treatment outcomes (Murray, 2006; Zhou et al., 2009). Inter-individual differences in enzymatic capacity may be related to the speed of breakdown of a medicine in the body, and slower or faster metabolization may be associated with worse treatment outcomes (Ingelman-Sundberg, 2005). For drugs prescribed in psychosis, the *CYP2D6* enzyme has been described as the most important metabolizing gene (Zhang and Malhotra, 2011). The *CYP2D6* gene is located on chromosome 22q13.1 and is characterized by its high complexity (Gaedigk, 2013). Thus far, more than 100 genetic variants of *CYP2D6* have been described, and many are associated with reduced or absent enzyme activity (PharmVar, 2023, accessed 23 November 2023). As a result, several institutions such as the Dutch Pharmacogenetics Working Group (DPWG) (Beunk et al., 2023) and Clinical Pharmacogenetics Implementation Consortium (CPIC) (CPIC, 2023, accessed 23 November 2023) have published, and continue to develop, dosing guidelines to account for these variations. Not all drugs intended to treat psychosis are metabolized through *CYP2D6*, however. The DPWG guidelines include aripiprazole, brexpiprazole, haloperidol, pimozide, risperidone, and zuclopenthixol for *CYP2D6*, and quetiapine for *CYP3A4* (Beunk et al., 2023). Although other medication may thus not be affected by individual variance in *CYP2D6* metabolization, this enzyme remains the strongest candidate for PGx-guided treatment for psychopharmaceutics and psychosis medication (van Westrhenen et al., 2021).

To improve standardization, Gaedigk et al. (2008, 2018) have developed the activity score (AS) system where each variant, represented by a star (*) allele, is assigned a score representing its activity. Carriers of two null alleles (AS = 0) are considered “poor metabolizers” (PM), individuals with one null allele and one reduced function allele, or two reduced function alleles (AS = 0.5) are classified as “intermediate metabolizers” (IM), a “normal metabolizer” (NM) (AS = 1–2) is someone with one reduced function and one normal or two normal function alleles, and when a person has more than two normal function alleles due to copy number variants, they are an “ultrarapid metabolizer” (UM) (AS > 2) (Gaedigk et al., 2008, 2018). Previous studies have associated the PM phenotype with increased risk of adverse effects such as weight gain and extrapyramidal symptom, or overall discontinuation of risperidone (de Leon et al., 2005; Ito et al., 2018; Jürgens et al., 2022; Kobylecki et al., 2009; Wannasuphoprasit et al., 2022). However, others have found no association between *CYP2D6* phenotype and treatment outcome (Austin-Zimmerman et al., 2021; Calafato et al., 2020; Nasyrova et al., 2023; Plesnicčar et al., 2006; Scordo et al., 2000; Walden et al., 2019).

Conflicting results may be explained by the lack of adequately sized randomized clinical trials examining PGx in drugs for psychosis, or by ignorance of challenges inherent to naturalistic cohort studies, such as retrospective and self-report bias (Lira et al., 2022; Rosenman et al., 2011). The first potential limitation would be resolved through larger clinical trials, such as the PSY-PGx project currently being conducted (Pelgrim et al., 2024). The other may be improved through the consideration of potential underlying factors contributing to these mixed results in new study designs. For example, patients included in retrospective designs may vary in the amount of time they have been in treatment. This can affect the treatment regime, since certain medications may not be preferred for treatment-naive patients (Hui et al., 2019; van Alphen et al., 2012).

Existing PGx guidelines suggest mostly alternative medication or alterations of dose compared to a standard dose of a specific medication, based on the idea that PM individuals have an increased risk of side effects at the standard dose. In clinical practice, if all patients were treated with this same dose, PM individuals would be expected to report worse outcomes. However, patients in cohort studies often have been in treatment for an extended amount of time. They may have seen a physician or psychiatrist more often and have had their treatment regime adapted according to their subjective experience with a medication. Consequently, they may have already been placed on a specific treatment choice or dose tailored to them after trying other medications. If this is the case, it may be expected that PM individuals are treated with lower doses of drugs for psychosis compared to NM or UM individuals. Ignoring this in retrospective analyses or prospective study designs may mask potential between-phenotype group differences in treatment outcomes due to PM patients reporting less side effects, rather than more, as they are already treated with a lower dose. Since most healthcare professionals adapt medication based on patient reports on effectiveness and tolerance, it could be hypothesized that treatment is therefore unconsciously adapted to the patient’s *CYP2D6* phenotype. This may be even more likely if the patient is not treatment-naive and has experienced adverse reactions to standard doses before.

Some evidence exists supporting this hypothesis. Previously, Mas et al. (2012, 2017) demonstrated that clinicians prescribed lower doses of risperidone for PM patients with first episode psychosis, without information on patients’ *CYP2D6* activity. However, to the best of our knowledge, this has not been replicated elsewhere. Additionally, their samples were limited in size and primarily consisted of psychiatric inpatients, which may not generalize to the whole population of users of drugs for psychosis. To overcome this limitation and further contribute to the field of PGx in drugs of psychosis, we replicated the study by Mas et al. (2012, 2017) in a naturalistic sample of people with psychotic disorders.

In this study, we examined patients with a psychotic disorder under pharmacological treatment in a longitudinal Dutch cohort. Patients were eligible for inclusion if they were already diagnosed with a non-affective psychotic disorder, and were genotyped a posteriori, during the “Genetic Risk and Outcome of Psychosis” (GROUP) study for which *CYP2D6* phenotype was not a main outcome. Thus, their prescribing healthcare professionals were not aware of their *CYP2D6* metabolization type. In this large and diverse cohort, we investigated whether the chlorpromazine-equivalent dose of 10 medications (risperidone, olanzapine, quetiapine, clozapine, haloperidol, aripiprazole, flupentixol, penfluridol, zuclopentixol, and amisulpride) was related to *CYP2D6*-predicted phenotype. Additionally, we evaluated which patient-related factors contributed to prescribed dosage. Based on the previous outcomes of Mas et al. (2012, 2017), we hypothesize that PM individuals report using lower doses of their treatment medication, and that *CYP2D6* phenotype contributes to dosage. In addition to replicating and expanding on existing work, this study may further contribute to the field of PGx by investigating a potential hypothesis contributing to the lack of conclusive results in PGx studies examining *CYP2D6* and treatment outcomes of drugs for psychosis.

## Method

### Study design and participants

Data from the GROUP cohort were used to perform a retrospective analysis. The GROUP consortium and study protocol has been described in detail elsewhere (Korver et al., 2012). In short, patients were identified through clinicians at local psychosis departments or academic institutions in the Netherlands and Flemish Belgium. Both in- and outpatients were eligible. Patients were eligible for inclusion in the GROUP study if: (1) they were between 16 and 50 years of age; (2) diagnosed with non-affective psychotic disorder according to the Diagnostic and Statistical Manual of Mental Disorders, Fourth Edition (DSM-IV) (American Psychiatric Association, 2000), and with first contact with mental health care within the previous 10 years; (3) had good command of the Dutch language; and (4) were able and willing to provide written informed consent. The study was conducted in accordance with the Declaration of Helsinki (2013). The protocol was centrally approved by the Ethical Review Board of the University Medical Center Utrecht (protocol code 04/003, May 2004), as well as by each local review board per participating center. Written informed consent was obtained from all participants before the first assessment, and after providing verbal and written information about the study. Each participant was given a unique research identification number, to maintain confidentiality. This number does not include any information that could lead to the identification of the participant. All personal data associated with the number were stored locally by each center.

In the current study, patients who were prescribed at least one treatment drug for psychosis at measurement 1 (baseline), and for whom genotype-predicted phenotype of *CYP2D6* was available, were selected. There were no further criteria. *CYP2D6* was chosen as the PGx gene of interest due to the relatively large number of medications for which it appears to be actionable in the treatment of psychosis (Beunk et al., 2023). Prior to analysis, patients with a reported chlorpromazine-equivalent dose of the main treatment drug below 25 mg or above 1000 mg per day were removed, to avoid influence of extreme doses. A chlorpromazine-equivalent dose of 1000 mg exceeds the maximum dose recommended for most drugs for psychosis, and 25 mg would be extremely low and likely represents an entry mistake (Lee and Remington, 2014; Woods, 2003). Chlorpromazine-equivalent dose, rather than medication-specific dose, was used to aid comparability and to account for variability in recorded units of medication-specific dose.

### Genotyping and phenotype prediction of *CYP2D6*

A tube of 20 ml of blood was collected from each subject. DNA extraction was performed from peripheral blood lymphocytes at the University Medical Center Utrecht. The complete GROUP sample was utilized in the genotyping pipeline. PLINK V1.9 was used to perform quality control procedures (Purcell et al., 2007; Purcell and Chang). For detailed information on genotyping and quality control, refer to (Sandhu et al., 2023). The full GROUP cohort, on which the genotyping was performed, included 2818 individuals (patients, siblings, parents, and healthy controls). Only a subset was included in this study, consisting of patients taking a treatment drug for psychosis at baseline. A total of 570,038 single-nucleotide variants (SNVs) were generated on a customized Illumina Institute of Psychological Medicine and Clinical Neurology chip array. Following extensive quality control, non-genotyped variants were imputed using the 1000 Genome Phase 3 (V5) reference panel using the Michigan Imputation Server (Das et al., 2016). A post-imputation filter of *R*^2^ > 0.5 was applied to ensure the inclusion of high-quality variants only.

The python-based bioinformatics tool Stargazer was used to predict AS and metabolizer phenotype of *CYP2D6* (Lee et al., 2019a, 2019b). Following imputation, chromosome 22 data were used to identify *CYP2D6* star alleles (haplotypes), ASs, and phenotype classification for each individual. Phenotype classifications were cross-checked with the PharmVar genotype-phenotype translation tables and the Pharmacogenomics Knowledgebase (PharmGKB; PharmVar, 2022), as described in detail by Sandhu et al. (2023).

### *CYP2D6* inhibitor identification

Information on the medication reported to be used by the patient was used to identify *CYP2D6* inhibitors and their associated strength according to the table describing CYP inhibitors by (Flockhart et al., 2021). See Table S1 for an overview of inhibitors and their associated strength.

### Measures

Positive And Negative Syndrome Scale (PANSS): The PANSS was administered by trained researchers to measure symptom severity, as it assesses both positive and negative symptoms of schizophrenia combining the Brief Psychiatric Rating Scale and Psychopathology Rating Schedule in a 30-item questionnaire (Kay et al., 1987). Each item is rated on a 7-point scale from 1 (*absent*) to 7 (*extreme*). General psychopathology was also assessed. Scale-specific items were summed to calculate the positive and negative symptom (each 7 items; minimum score 7, maximum score 49) and general psychopathology (16 items; minimum score 16, maximum score 112) subscale scores. These scores have been demonstrated to have strong reliability, validity, and sensitivity (Kay et al., 1987; Leucht et al., 2005; Peralta and Cuesta, 1994).

### Covariates

Age, sex, *CYP2D6* inhibitor use, *CYP2D6* inhibitor strength, smoking status, and illness duration in years were included as covariates. Although a known covariate, BMI was not available and was thus not included.

### Statistical analysis

Statistical analyses were performed using R version 4.2.3 (R Core Team, 2023). Kruskal–Wallis between-group tests were used to examine differences in chlorpromazine-equivalent dose between *CYP2D6* phenotype groups. To investigate the relationship between chlorpromazine-equivalent dose and hypothesized variables of interest, Spearman’s correlation was used. Variables of interest were age, sex, *CYP2D6* inhibitor use, PANSS positive score, PANSS negative score, PANSS general psychopathology score, smoking status, and illness duration. General linear models were used to predict chlorpromazine-equivalent dose. The final model was selected through stepwise backward reduction and comparison of all models using the Akaike information criterion (AIC) (Bozdogan, 1987) and *R*^2^ (Colin Cameron and Windmeijer, 1997). The *R* “stats” package provided the following functions to perform these tests: *kruskal.test(), cor.test()*, and *glm()* (R Core Team, 2023). To calculate and compare corrected AIC (AICc), the *aictab()* function from the AICcmodavg package was used (Mazerolle, 2023).

As an exploratory analysis, the statistical analyses were repeated for each drug separately and for a group of PGx-actionable drugs for which *CYP2D6*-specific DPWG guidelines are available (aripiprazole, brexpiprazole, haloperidol, pimozide, risperidone, and zuclopenthixol) (Beunk et al., 2023).

*p* < 0.05 was considered significant. GROUP release number 8.0 was used for the current analysis.

## Results

### Patient characteristics

Patients who were prescribed at least one treatment drug for psychosis at measurement 1 (baseline) and for whom genotype-predicted phenotype of *CYP2D6* was available were selected. *N* = 418 patients were included (79.7% male, mean age 27 years). Most patients were diagnosed with schizophrenia (86.4%), followed by other psychotic disorders (12.4%). For two patients (0.5%), diagnosis was unknown, but they were taking a treatment drug for psychosis and were thus included. Seventeen patients (4.1%) were genotype-predicted PMs, whereas 167 patients (40%) and 228 patients (54.6%) were predicted IMs and NMs, respectively. For six patients (1.4%), genotype could not be predicted. The UM phenotype could not be predicted due to limitation in the genome-wide association (GWAS) panel. A Chi-square test confirmed that the phenotype distribution in this sample was not different than expected in a European population, as previously described in existing literature (Gaedigk et al., 2017; *p* = 0.29). Table 1 provides an overview of patient characteristics.

**Table 1.** Demographic variables of the complete sample.

Demographic variables	*N*	%
Patients	418	100
Sex, male/female	333/85	79.7/20.3
Average age in years	27.25 (±7.1)	
Average duration of illness in years	4.4 (±3.4)	
Number of psychotic episodes	1.7 (±1.1)	
Duration of psychosis untreated before first treatment drug for psychosis (in months)	10.85 (±24.98)	
PM	18.7 (±56.1)	
IM	12.2 (±24.9)	
NM	9.29 (±21.3)	
Unknown	8.4 (±10.4)	
Ethnicity		
White	344	82.3
Moroccan	13	3.1
Turkish	12	2.9
Other	10	2.4
Mixed	35	8.4
Unknown	4	1
Diagnosis		
Schizophrenia	361	86.4
Bipolar disorder	2	0.5
Psychotic disorders	52	12.4
Substance use disorder	1	0.2
Unspecified	2	0.5
Smoking status		
Smoker	261	62.4
Non-smoker	150	35.9
Unknown	7	1.7
Main treatment drug		
Olanzapine	125	29.9
Risperidone	112	26.8
Clozapine	60	14.4
Aripiprazole	50	12
Quetiapine	28	6.7
Haloperidol	16	3.8
Flupentixol	11	2.6
Amisulpride	8	1.9
Zuclopentixol	3	0.7
Penfluridol	3	0.7
Perphenazine	1	0.2
Sulpiride	1	0.2
*CYP2D6* inhibitor status		
Use of one or more inhibitors as defined by Flockhart et al. (2021)	104	24.9
None	314	75.1
*CYP2D6* inhibitor strength		
Weak	33	31.7
Strong	71	68.3
*CYP2D6* genotype predicted phenotype		
PM	17	4.1
IM	167	40
NM	228	54.5
Unknown	6	1.4
Chlorpromazine-equivalent dose of main treatment drug (in mg)	360 (±193)	
Chlorpromazine-equivalent dose of main treatment drug (in mg) per *CYP2D6* predicted phenotype		
PM	331 (±209)	
IM	342 (±174)	
NM	373 (±137)	
Unknown	447 (±137)	

Average variables are presented with standard deviations.

PM: poor metabolizer; IM: intermediate metabolizer; NM: normal metabolizer.

### Between-group comparison

**Figure 1.**
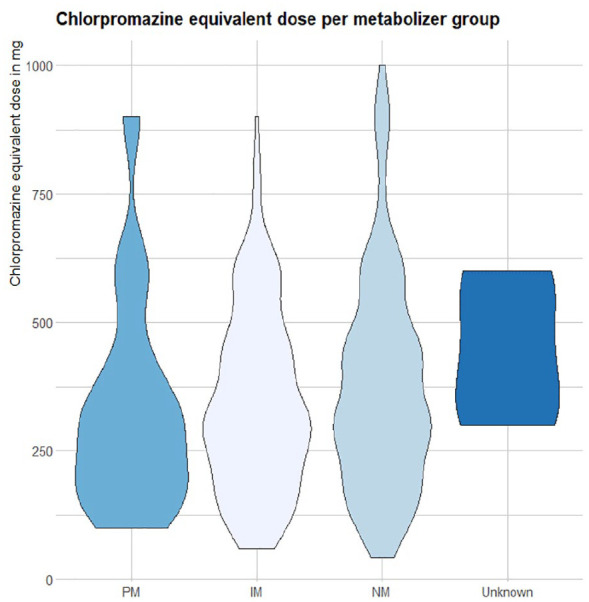
Distribution of reported chlorpromazine-equivalent dose of treatment drug between metabolizer groups. PM: poor metabolizer; IM: intermediate metabolizer; NM: normal metabolizer.

A Kruskal–Wallis test indicated no difference of chlorpromazine-equivalent dose between phenotype groups (*p* = 0.231). Figure 1 illustrates the distribution of chlorpromazine-equivalent dose between metabolizer groups.

### Correlation

When all treatment drugs were considered together, *CYP2D6* phenotype was not found to be correlated with chlorpromazine-equivalent dose (*p* = 0.3, Rho = 0.05). Sex, smoking status, illness duration in years, and all three PANSS scales were significantly correlated with chlorpromazine-equivalent dose (*p* < 0.01 for all except illness duration in years, which was significant at *p* = 0.03). All correlations were weak and in a positive direction, except for sex, which was negative, suggesting that women are prescribed lower doses. Age and *CYP2D6* inhibitor use were not found to be significantly associated with chlorpromazine-equivalent dose. Similar results were found when only considering PGx actionable drugs (*p* = 0.82, Rho = 0.02 for the correlation with *CYP2D6* phenotype) or risperidone only (*p* = 0.34, Rho = 0.09). Figures S1 and S2 in the Supplemental material provide an overview of the correlations of chlorpromazine-equivalent dose with all other variables, as well as variables with each other.

**Figure 2.**
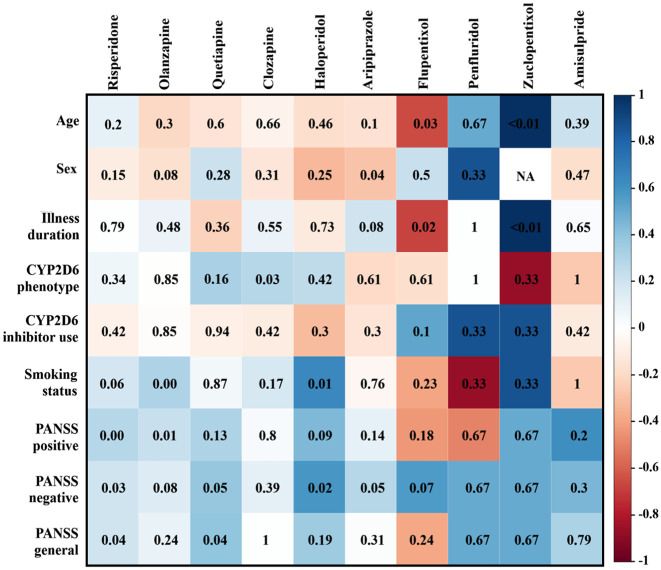
Spearman correlations between dose of drug and other variables. It should be noted that there is no correlation between zuclopentixol and sex, since this could not be calculated. In this figure, negative through positive correlations are presented in a color gradient. The *p*-value for the correlation with a drug and measure is depicted inside the box.

*CYP2D6* phenotype was significantly correlated with dose when examining drugs separately, specifically with clozapine (*p* = 0.03, Rho = 0.28). Age was correlated with flupentixol (*p* = 0.03, Rho = −0.66) and zuclopentixol dose (*p* < 0.001, Rho = 1), although it should be noted that only three patients were treated with zuclopentixol. Female sex was negatively, but weakly, associated with aripiprazole (*p* = 0.04, Rho = −0.29) and illness duration was only associated with zuclopentixol (*p* < 0.001, Rho = 1). A weak correlation was also found between chlorpromazine-equivalent dose of risperidone (*p* < 0.01, Rho = 0.28) and olanzapine (*p* = 0.01, Rho = 0.23), and the PANSS positive scale. In fact, risperidone dose was weakly correlated with all three PANSS scales (positive: *p* < 0.01, Rho = 0.28, negative: *p* = 0.03, Rho = 0.21, general: *p* = 0.04, Rho = 0.2). Quetiapine dose was associated with the PANSS general scale (*p* = 0.04, Rho = 0.4), and haloperidol (*p* = 0.02, Rho = 0.58) and aripiprazole (*p* = 0.05, Rho = 0.28) were correlated with the PANSS negative scale. Last, smoking status was only associated with the chlorpromazine-equivalent dose of olanzapine (*p* < 0.01, Rho = 0.3) and haloperidol (*p* = 0.01, Rho = 0.63). Figure 2 provides an overview of all correlation findings for each drug.

### General linear model

Prior to model selection, multicollinearity between predictors was checked using variance inflation factor (VIF). Multicollinearity may be present when VIF > 5; thus, this was used as a cut-off score (Kim, 2019). When both included in the model, *CYP2D6* inhibitor status and *CYP2D6* inhibitor strength exceeded a VIF score of 5. Therefore, *CYP2D6* inhibitor status was removed as predictor. *CYP2D6* inhibitor strength remained because it was expected to provide more information as a predictor. Following removal, no VIF score was higher than 2.7 (PANSS general score).

The final model for all treatment drugs together (*R*^2^ = 0.11, AICc = 5319.5) included the following significant predictors: all PANSS scales (general: *p* = 0.044, β = −80.3, 95% CI = −157.6 to −2.9; negative: *p* < 0.01, β = 53.9, 95% CI = 16.3–91.4; positive: *p* < 0.01, β = 70.6, 95% CI = 27.4–113.8) and sex (*p* = 0.038, β = −66.6, CI = −129.1 to −4.1). Additionally, *CYP2D6* phenotype, *CYP2D6* inhibitor strength, and illness duration in years were included, but none were significant (*p* = 0.33, *p* = 0.19 and *p* = 0.17, respectively). In the PGx-actionable subset, the best fitting model (*R*^2^ = 0.16, AICc = 2269.6) was found to be the complete model. Of the included variables, the same predictors were found to be significant: all PANSS scales (general: *p* = 0.028, β = −90.1, 95% CI = −169.7 to −10.5; negative: *p* < 0.01, β = 55.7, 95% CI = 17.5–93.8; positive: *p* < 0.01, β = 73.6, 95% CI = 28.8–118.3) and sex (*p* = 0.034, β = −71.8, 95% CI = −137.5 to −6.0). All other included predictors were not significant (*CYP2D6* phenotype: *p* = 0.27; age: *p* = 0.42; *CYP2D6* inhibitor strength: *p* = 0.19; smoking status: *p* = 0.92; illness duration: *p* = 0.41). The direction of the effect was the same in both samples, where lower scores on the PANSS general scale, higher scores on the PANSS negative and positive scales, and male sex predicted higher dosage.

Due to the small sample sizes per treatment drug, a prediction model was not feasible for each medication separately. Therefore, only for the drugs for which the sample size was 50 or larger, did we calculate a prediction model. Table 2 provides the best fitting models, with estimate and confidence interval of the estimate for those predictors that reached significance. It should be noted that the prediction direction is the same as in the combined and PGx-actionable samples. Age, which was not previously found to contribute, was significant in the model for olanzapine, suggesting increased age was associated with lower dosage. *CYP2D6* phenotype was significant for prediction of clozapine, and related to higher dose at NM versus IM, and IM versus PM groups. This model predicted PMs to be administered the lowest chlorpromazine-equivalent dose of clozapine.

**Table 2.** Significant predictors of the final reduced general linear models for dosage of olanzapine, risperidone, clozapine, and aripiprazole following stepwise backward removal. Final models were chosen based on AICc. Predictors with p < 0.05 are in italic and have effect size and 95% confidence interval provided. PANSS: Positive and Negative Symptom Scale.

Olanzapine (*N* = 125)	Risperidone (*N* = 112)	Clozapine (*N* = 60)	Aripiprazole (*N* = 50)
*R*^2^: 0.16, AICc: 1580.8	*R*^2^: 0.1, AICc: 1405.2	*R*^2^: 0.18, AICc: 803.5	*R*^2^: 0.38, AICc: 620.2
Age (*p* = 0.02, β = −5.9, CI = −10.9; −1.0)	Age (*p* = 0.19)	*CYP2D6* phenotype (*p* = 0.03, β = 110.5, CI = 13.4; 207.6)	*CYP2D6* phenotype (*p* = 0.28)
PANSS positive (*p* = 0.049, β = 45.4, CI = 0.5; 90.2)	Sex (*p* = 0.36)	Age (*p* = 0.11)	Sex (*p* = 0.19)
Smoking status (*p* = 0.067)	PANSS positive (*p* = 0.068)	Sex (*p* = 0.18)	*CYP2D6* inhibitor strength (*p* = 0.31)
Illness duration (*p* = 0.03, β = 10.9, CI = 1.0; 20.8)	PANSS negative (*p* = 0.13)	PANSS negative (*p* = 0.59)	PANSS general (*p* = 0.04, β = −224.6, CI = −366.8; −81.3)
	Smoking status (*p* = 0.51)	Smoking status (*p* = 0.29)	PANSS positive (*p* = 0.082)
			PANSS negative (*p* = 0.007, β = 113.1, CI = 34.8; 191.31)
			Smoking status (*p* = 0.38)
			Illness duration (*p* = 0.058)

## Discussion

The results of our analysis show that there is no correlation between *CYP2D6* phenotype and chlorpromazine-equivalent dose when all drugs for psychosis are considered together, nor for only those for which PGx guidelines are available (Beunk et al., 2023).

To our knowledge, this is the first study to examine the relationship of *CYP2D6* phenotype and dosage of any drug for psychosis in a naturalistic sample. Two prior studies have examined the relationship of CYP2D6 phenotype and risperidone dosage. In Mas et al. (2012) reported a strong correlation between *CYP2D6* metabolizer status and risperidone dosage, although the observed differences in dosage were modest between phenotype groups. In 2017, this was replicated in a different cohort of patients, who were receiving medication for less than 1 year. Similar results were found regarding intuitive adjustment of dosage by clinicians according to *CYP2D6* phenotype (Mas et al., 2017). In both studies, reduced metabolizer status (IM or PM) was associated with lower dosage of risperidone. We aimed to replicate and extend this work by investigating a larger, more diverse sample including multiple medications. Considering that Mas et al. (2012, 2017) investigated risperidone dose only, we have replicated this by stratifying the sample by specific medication and repeating the analysis. We again found no correlation between risperidone dose and *CYP2D6* phenotype in our sample, nor was there a significant correlation in the PGx-actionable subset. Thus, our hypothesis that medication dose was unintentionally already adapted to the patients’ *CYP2D6* phenotype was rejected.

We aimed to investigate whether this could be explained by the hypothesis that “intuitive” dosing of clinicians, who would adapt pharmacological treatment to *CYP2D6* activity without existing knowledge of the patient’s genotype, masks the influence of *CYP2D6* phenotype on treatment outcome and adverse reactions. The finding that medication dose was not lower for PM individuals in this sample suggests that this is likely not the case. This may strengthen existing studies where this was not controlled for. In a previous study in the same cohort, we demonstrated a mixed association of *CYP2D6* phenotype or phenoconversion-corrected phenotype with side effects, subjective well-being, or PANSS scores, with some effects on specific outcomes for risperidone, olanzapine, and clozapine (de Brabander et al., 2024). The results of other studies on this topic have been similarly divergent, as some research groups report worse outcomes for PM/IM compared to NM individuals (e.g., Al Maruf et al., 2021; Facal et al., 2023; de Leon et al., 2005; Ito et al., 2018; Jürgens et al., 2022; Jukic et al., 2019; Kobylecki et al., 2009; Milosavljević et al., 2021; Scordo et al., 2000; Walden et al., 2019), yet others find no relationship (e.g., Armstrong et al., 1997; Austin-Zimmerman et al., 2021; Beunk et al., 2023; Calafato et al., 2020; Nasyrova et al., 2023; Rudå et al., 2021; Plesnicčar et al., 2006; Wannasuphoprasit et al., 2022). Together, this may suggest a limited effect of *CYP2D6* or only in certain cases, for example in patients with currently unknown predisposing factors such as affective temperaments increasing their risk of side effects (de Aguiar Ferreira et al., 2014; Pompili et al., 2012, 2018).

Our results contrast with the original studies by Mas et al. (2012, 2017), as we could not reproduce similar findings entirely. A probable explanation is the inclusion of drugs other than risperidone in our study, though several other factors may have contributed. First, our sample included less PM individuals (7.1%–9.9% vs 4.1%). Of these PM patients (*n* = 17), half were taking a primary medication for psychosis metabolized by CYP2D6 (risperidone: *n* = 5; haloperidol: *n* = 1; aripiprazole: *n* = 3). As such, only about a third of PM patients were treated with risperidone compared to all patients in the previous studies. However, we attempted to overcome this by repeating the analysis in a PGx-actionable and risperidone-only subset, although the percentage of PM patients remained low in both (5% and 4.5%, respectively).

Furthermore, in the first study by Mas et al. (2012), risperidone prescription was likely more systematically regulated, due to the inpatient status of the participants. In the current sample, patients reported the use and dose of their main treatment drug themselves and were not restricted to monotherapy with a specific medication. This is a similar design as Mas et al. (2017), where a subset of the “Phenotype–genotype and environmental interaction. Application of a predictive model in first psychotic episodes” (PEPs) study was analyzed. Likewise to GROUP, in this naturalistic cohort there were no guidelines for treatment (Bernardo et al., 2013; Korver et al., 2012). In both studies, data collection regarding medication use through interviews and self-reports may be at increased risk for documentation errors or data inaccuracies. Cognitive problems associated with schizophrenia and psychotic disorders may further complicate recall and reporting of medication use and adherence (Birnbaum and Sharif, 2008).

A notable difference between our study and that of Mas et al. (2017) is how medication adherence was assessed. In the PEPs study, blood plasma levels of the treatment drug were measured (Bernardo et al., 2013). Although this is an indirect measure of adherence, it provides an advantage over the current sample. In GROUP, medication compliance was estimated by the treating physician rather than through blood plasma levels. Although treatment adherence should not affect the potential relationship between medication dose and CYP2D6 phenotype directly, it may have influenced medication prescription. Challenges with treatment adherence have previously been described in users of drugs for psychosis (Acosta et al., 2012; Guo et al., 2023), and factors such as fear of potential side effects and polypharmacy can increase nonadherence (Brown and Bussell, 2011; Jimmy and Jose, 2011). Experience of side effects may also be associated with increased levels of non-adherence, although this is appears to be only a weak predictor of adherence in drugs for psychosis (Dibonaventura et al., 2012; El Abdellati et al., 2020; Lieslehto et al., 2022). If not communicated to the prescribing physician, the treatment regime may remain sub-optimal, and patients are continued to be prescribed a dose that is possibly too high or too low. Nonadherence could thus potentially contribute to incorrect self-report of medication use and/or dose.

Although we did not confirm the previous findings regarding *CYP2D6* phenotype, our results did partly agree with Mas et al. (2017) regarding other factors. Namely, we found that risperidone dose was weakly, but significantly, correlated with all three PANSS scales. These scales were also positively correlated with dosage in the complete sample, as well as the PGx actionable subset, although correlation strength remained weak. These three scales were also most often found to correlate with dosage for other drugs, suggesting that at higher scores of the PANSS, higher chlorpromazine-equivalent dose would be prescribed. These findings may not be unanticipated, considering that higher scores of the PANSS are associated with increased impairment related to psychotic symptoms (Leucht et al., 2005). Sex has also previously demonstrated to be related to dosage, as women are often prescribed lower doses due to for example, differences in body composition, such as smaller organs and increased fatty tissue, different gastric emptying and intestinal motility, and drug metabolism (Brand et al., 2022; Seeman, 2004). Similarly, smoking status has been associated previously with increased dose, which may be due to the effect of smoking on *CYP1A2* activity, an enzyme also involved in the metabolism of certain drugs for psychosis, such as olanzapine and clozapine (Sagud et al., 2009; Tsuda et al., 2014). Thus, our results are in line with existing literature on patient factors affecting dose. Illness duration was also found to be positively, albeit weakly, associated with dose. A possible explanation is that patients may be in need of a progressively higher dose to continue to manage symptoms over time, although previous research suggests that increasing dose for maintenance does not lead to improved outcomes (Leucht et al., 2021). Rather, this correlation may represent treatment resistant patients, as the PANSS positive scale was also significantly correlated with illness duration and dose. Since acute treatment drug efficacy is greater for positive symptoms, a combination of higher PANSS positive score and longer illness duration may suggest a lack of treatment response and potential treatment resistance (Haddad and Correll, 2018).

Interestingly, clozapine chlorpromazine-equivalent dose was found to be (weakly) correlated with *CYP2D6* phenotype. *CYP2D6* phenotype also significantly predicted chlorpromazine-equivalent dose in the general linear model, where decreased enzyme function was associated with decreased dose. Notably, clozapine is not included in the DPWG guidelines due to lack of evidence for a PGx effect. It is mainly metabolized through *CYP1A2*, with a minor role for *CYP2D6* (Thorn et al., 2018). This relationship may be explained, at least in part, by the status of clozapine as pharmacological choice for treatment-resistant psychosis in The Netherlands. Prescription guidelines suggest the use of clozapine only after treatment failure with two previously prescribed drugs for psychosis (van Alphen et al., 2012). PM and IM individuals may experience adverse reactions at increased frequency or severity compared to NM patients and, therefore, may be at higher risk of treatment failure and are more likely to be prescribed clozapine, at which stage their history with adverse reactions may warrant clinicians to remain conservative in changing or increasing after initiation. Although this could suggest that *CYP2D6* phenotype is associated with treatment resistance, evidence for this hypothesis is limited (Aitchison et al., 1999; Okhuijsen-Pfeifer et al., 2022; Shin et al., 2021; Sosin et al., 2019). It may be of interest to investigate this in future studies.

There are several limitations that should be considered. First, *R*^2^ for all general linear models was low (*R*^2^ < 0.2), with exception of that of aripiprazole (*R*^2^ = 0.38). This may be due to the higher number of included variables, as *R*^2^ tends to increase in larger models (Cameron and Windmeijer, 1996; Nakagawa and Schielzeth, 2013). Second, there was no reliable information available on how long patients were treated with their reported treatment drug. We also did not know with certainty if the current treatment was the patient’s first treatment, and as such we could not calculate the duration of treatment with the available information. In the Netherlands, for most drugs for psychosis, a patient starts with a specific dose, which is altered in increments according to the experience of the patient (van Alphen et al., 2012). Thus, someone who recently started pharmacological treatment may be on such a standard starting dose, rather than on their optimal regime. Our hypothesis that non-NM patients would be prescribed higher or lower doses (for UM or PM phenotype, respectively) assumes that patients have had previous negative experiences with the standard dose. We could not investigate whether this may have affected our results. Third, medication type and dosage were recorded through self-report during patient interviews. Recall bias or report errors may have occurred. During the measurement, participants may not have had their medication prescriptions on hand and would have had to recall their medication name and dose. Previous studies have suggested potential underreporting of medication use in self-reports during interviews (Garber et al., 2004; Hafferty et al., 2018; West et al., 1995). Fourth, we were not able to assess dosage for UMs, due to limitations of the GWAS panel. Finally, although the overall sample size was large, the sample size for the DPWG-only subset and for each drug was small. As a result, some subsets included few to no PM patients. Additionally, statistical power will have suffered for the drug-stratified correlation analysis.

Despite these limitations, this study presents a relatively large and diverse group of real-world patients treated with a variety of drugs for psychosis, which improves generalizability over previous studies. Considering the existing debate on the clinical utility of PGx in treatment of psychosis, investigations of potential underlying mechanisms of contradicting results may be valuable to aid interpretation of existing findings. Although our findings did not confirm our hypothesis regarding unconscious treatment optimization according to *CYP2D6* phenotype in this naturalistic sample and could thus not further explain why PGx research remains inconclusive, it may rather provide additional support for existing retrospective cohort PGx studies. *CYP2D6* phenotype may only have a limited or specific effect in treatment using drugs for psychosis and does not appear to affect medication choice or dose in practice for most patients.

## Supplemental Material

sj-docx-1-jop-10.1177_02698811241279022 – Supplemental material for Unidentified CYP2D6 genotype does not affect pharmacological treatment for patients with first episode psychosis Supplemental material, sj-docx-1-jop-10.1177_02698811241279022 for Unidentified CYP2D6 genotype does not affect pharmacological treatment for patients with first episode psychosis by Emma Y De Brabander, Thérèse van Amelsvoort and Roos van Westrhenen; GROUP Investigators in Journal of Psychopharmacology
