# Supplementary material for: Unidentified CYP2D6 genotype does not affect pharmacological treatment for patients with first episode psychosis
Source: J Psychopharmacol. 2024 Sep 29;38(12):1111–21. doi: 10.1177/02698811241279022 (PMC11528939; doi:10.1177/02698811241279022)
Supplement: sj-docx-1-jop-10.1177_02698811241279022 – Supplemental material for Unidentified CYP2D6 genotype does not affect pharmacological treatment for patients with first episode psychosis [file sj-docx-1-jop-10.1177_02698811241279022.docx]

**Supplementary material**

| Weak | Moderate | Strong | Evidence on strength in-vitro only | Strength level under review |
| --- | --- | --- | --- | --- |
| Amiodarone | Abiraterone | Bupropion | Chlorpromazine | Methadone |
| Celecoxib | Cinacalcet | Fluoxetine | Clemastine | Midodrine |
| Cimetidine | Clobazam | Paroxetine | Cocaine | Panobinostat |
| Citalopram | Doxepin | Quinidine | Haloperidol | Promethazine |
| Clomipramine | Duloxetine |  | Hydroxyzine |  |
| Diphenhydramine | Halofantrine |  | Metoclopramide |  |
| Escitalopram | Lorcaserin |  | Perphenazine |  |
| Hydrochloroquine | Moclobemide |  | Ticlopidine |  |
| Levomepromazine | Rolapitant |  | Tripelennamide |  |
| Ritonavir | Terbinafine |  |  |  |
| Sertraline |  |  |  |  |
| Vermurafenib |  |  |  |  |

Table S1. Overview of CYP2D6 inhibitors. Adapted from Flockhart et al. (2021).


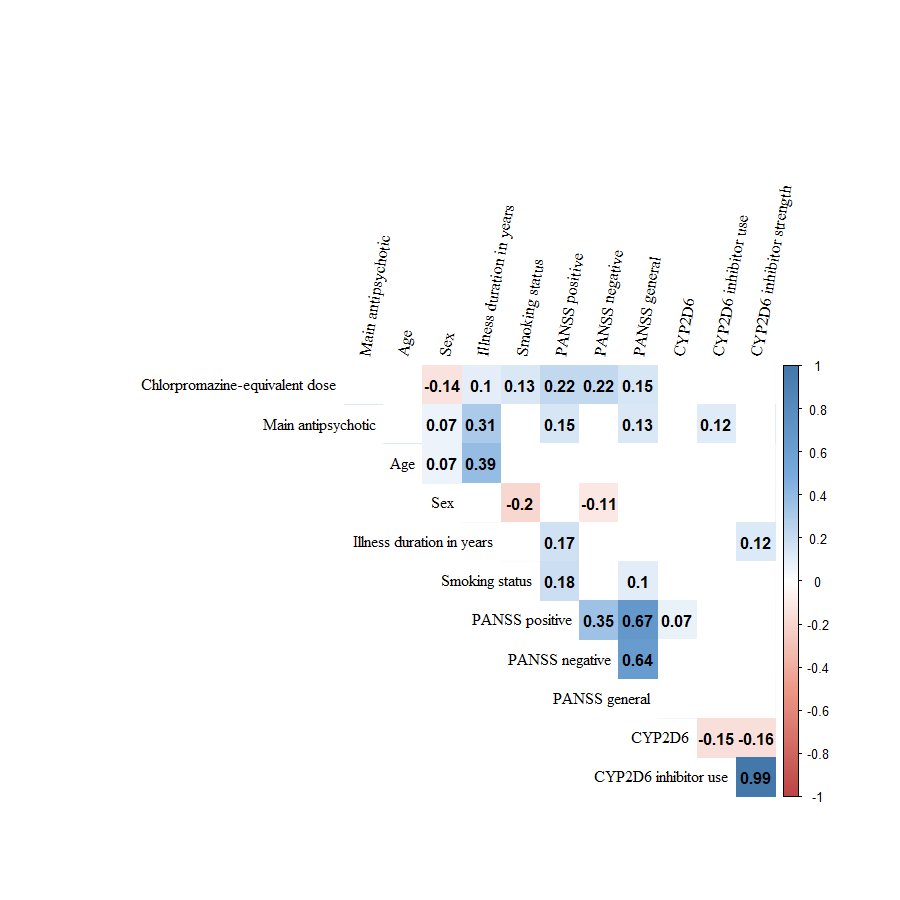


Figure S1. Spearman correlations between all variables for the complete sample including all treatment drugs. Number represents correlation strength. Blank squares were not significant.


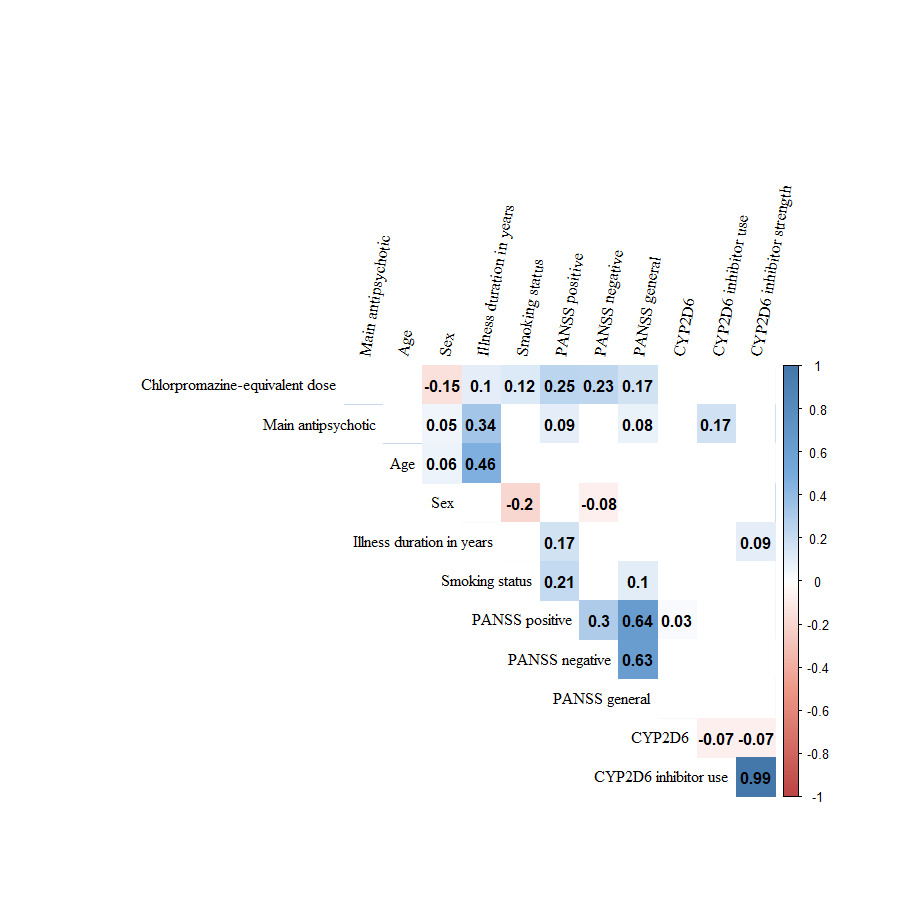


Figure S2. Spearman correlations between all variables for the sample including Dutch Pharmacogenetic Working Group (DPWG) drugs only. Number represents correlation strength. Blank squares were not significant.
